# Seasonal dynamic of ticks infesting cattle (*Bos indicus*) farms in two provinces in Cambodia

**DOI:** 10.1371/journal.pone.0320879

**Published:** 2025-04-16

**Authors:** Sony Yean, Didot B. Prasetyo, Theary Ren, Davy Krib, Saoya Sen, Bunthon Chea, Sothyra Tum, San Sorn, Samuth Sum, Sébastien Boyer

**Affiliations:** 1 Medical and Veterinary Entomology Unit, Institut Pasteur du Cambodge, Phnom Penh, Cambodia; 2 National Animal Health and Production Research Institute, General Directorate of Animal Health and Production, Phnom Penh, Cambodia; 3 Faculty of Veterinary Medicine, Royal University of Agriculture, Phnom Penh, Cambodia; Beni Suef University Faculty of Veterinary Medicine, EGYPT

## Abstract

Cattle tick infestations pose a significant threat to livestock health and productivity worldwide. These parasitic arthropods, feed on blood and may cause various diseases in cattle, such as anaplasmosis, babesiosis, ehrlichiosis, and theileriosis. The objective of this study is to understand the seasonal dynamic and distribution patterns of tick infesting cattle in Cambodia. A longitudinal tick survey was conducted from January to December 2023 in two cattle farms in the Takeo and Kampong Speu provinces. Ticks were collected directly from the animal hosts with fine-tip forceps or specialized tools such as tick twisters, and from the vegetation using a combination of dragging and flagging method. A total of 13,678 ticks were collected from 240 inspected cattle and 11,384 ticks were found from vegetation around the two farms. The most prevalent species was *Rhipicephalus microplus* complex (60.6%), while *R. haemaphysaloides* was recorded in small numbers (0.4%). Unidentified species represented 39% of the collected ticks, consisting of immature *Rhipicephalus* spp. In addition, one adult *Rhipicephalus linnaei*, 245 immatures of *Haemaphysalis* spp., and 11,138 larvae of *Rhipicephalus* spp. were collected using dragging and flagging methods. The results revealed significant seasonal differences in tick population dynamics across both provinces, with 60.24% in Kampong Speu and 57.09% in Takeo during the rainy season, compared to 39.76% and 42.91% during the dry season. Statistical analysis on questing activity showed no significant differences in tick density across different collection sites, times of the day and province. Our findings indicate a very high density of tick-borne pathogen vectors was observed on cattle in the two farms, which a poses potential risk to cattle productivity in Cambodia.

## Introduction

Ticks are recognized as the second most significant vectors, after mosquitoes, for transmitting a variety of pathogens to humans and animals, including viruses, bacteria, and protozoa [1–3]. Tick infestations on cattle pose a substantial risk to the health and productivity of livestock, particularly in tropical and subtropical regions [4–7]. Tick-borne diseases result in weight loss, decreased milk production [8,9], and increased costs for managing and treating affected animals. These issues can also lead to restrictions on animal movement between countries [10]. Approximately 80% of the global cattle population is affected by tick infestations, causing substantial economic losses [3,11–13]. An estimated USD 13.9–18.7 billion has been spent annually worldwide on economic losses and management of cattle tick infestation associated tick-borne diseases [14].

Ticks are obligate hematophagous organisms and play a crucial role as vectors in transmitting various pathogens that can affect livestock animals in Southeast Asia [15]. Common pathogens, such as those from the genus *Anaplasma*, *Babesia*, *Theileria* [15], and *Ehrlichia* [12,13,16,17] are frequently found in cattle in Southeast Asian countries, such as Indonesia [3], Thailand [16,18], Malaysia [19], and Vietnam [20]. *Rhipicephalus microplus*, a tick species from the Ixodidae family, is a major vector of tick-borne diseases affecting cattle. Originally native to India, it has spread to other Southeast Asian regions through cattle trade [21], becoming a significant threat to livestock due to its ability to transmit a variety of pathogens [3,22]. This species is the most prevalent tick infesting cattle and has been reported as the primary vector in Malaysia [8], Thailand [18,23], Indonesia [3], and Vietnam [24].

The prevalence of *Rhipicephalus microplus* highlights the importance of effective tick control strategies to mitigate its adverse effects on animal sustainability in the region. *Rhiphicephalus microplus* has a single-host life cycle throughout its parasitic phase which lasts about 23 days in development from larvae to nymph and then to adult. Once feeding is complete, engorged females detach from the host to lay eggs in the environment, marking the start of the non-parasitic phase and hatching of eggs, as well as the questing behavior of larvae to find a new host [25]. Understanding the ecology, behavior, and dynamics of ticks is crucial for developing targeted interventions to manage this vector and reduce its impact on livestock populations [26,27].

In Cambodia, *Bos indicus* cattle, commonly referred to as yellow cattle due to their distinctive yellowish coat color, are the predominant breed reared in the country. This local breed is crossed with Haryana and other breeds [28]. Due to limitations in studies and gaps in knowledge, no ticks or associated tick-borne diseases have been reported in cattle in Cambodia [29]. This study aims to investigate tick infestations on cattle and surrounding vegetation in farm settings in Cambodia, which is important for understanding tick biology and leading to a more effective control strategy.

## Methods

### Ethic statement

The study was approved and supported by the General Directorate of Animal Health and Production from the Ministry of Agriculture, Forestry and Fisheries of Cambodia, under permit No. 2981, signed on 1st October 2021. The study sites are located on both privately owned and government-owned land, but none are situated within protected areas. Ticks were collected exclusively from farmed cattle; no other animal species were sampled. During each collection effort, government veterinarians were present to oversee the cattle handling procedures. No invasive procedures have been performed and ticks were collected with minimal disturbance to the cattle.

### Study sites

**Fig 1:**
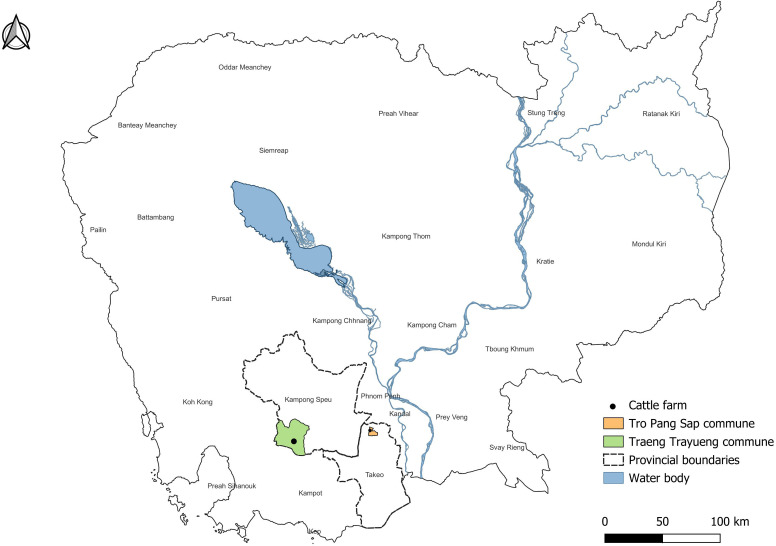
Tick collection sites at the cattle farm in Kampong Speu and Takeo province, Cambodia.

Two cattle farms located in Kampong Speu and Takeo provinces (Fig 1) were selected as study sites. These locations are about 76 km apart and have substantially different environmental conditions. Ecological variations, such as differences in altitude between the two sites, may affect temperature and humidity levels, which could influence tick presence and activity patterns. Both farms are also located near the capital, Phnom Penh, which provides logistical advantages for researchers in terms of accessibility and collaboration. The farm in Takeo is surrounded by vast grasslands and consists of 120 cattle, while the farm in Kampong Speu is surrounded by agricultural areas and has a total of 50 cattle. Cattle were periodically released for grazing on both farms. Tick inspections were conducted monthly from January to December 2023.

### Tick collection and morphological identification

In each farm, ten cattle were randomly selected every month in Kampong Speu and Takeo province. Additionally, some individual cattle were sampled multiple times throughout the month for tick collection. We conduct monthly interviews with farm owners and record the tick control measures implemented on the farms. However, it was noted that ivermectin was occasionally used to inject cattle, depending on the individual animal and not on a regular basis. Ticks were collected using direct sampling from cattle hosts and through dragging-flagging to collect questing ticks from vegetation. A team visually inspected seven predilection sites (head, ear, neck, back, abdomen including udder and scrotum, tail, and legs) on each cattle for 30 minutes per animal. Infesting ticks were removed from the host using fine-tip forceps or specialized tools like tick twisters, ensuring minimal discomfort to the animals. The collected ticks were stored in 15 ml conical tubes containing 70% ethanol and labeled according to the host and predilection site.

**Fig 2:**
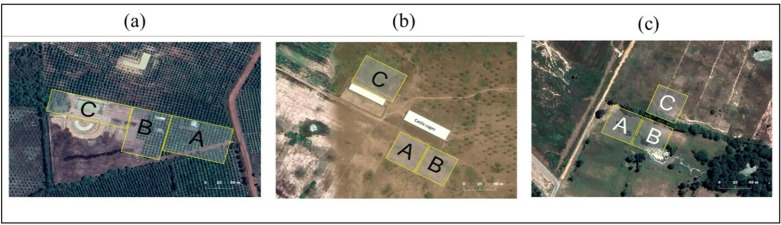
Dragging-Flagging sites (coded as A, B, C) for questing ticks from vegetation around cattle farm in (a) farm area in Kampong Speu, (b) farm and (c) grazing area in Takeo province.

Questing ticks were collected by dragging a 1 x 1m white cloth over vegetation in a designated area [30–32]. The dragging method involves moving the white cloth along a 50 m transect at a slow and steady pace, with stops every 10 meters to check for ticks [33]. Tick flagging was performed using a 40x60 cm white cloth attached to a 1.5 m pole, swiped over taller vegetation-like bushes, with frequent checks for ticks to prevent them from dropping off. Both dragging and flagging methods were employed by six collectors for up to 30 minutes, interchangeably depending on the landscape of the study sites. Collected ticks were removed from the cloths using masking tape, stored in individual Ziplock bags, and labeled by date, location, and time of collection. Dragging and flagging were conducted at three different times of the day corresponding to morning (7-8 am), midday (11-12 pm), and early evening (5-6 pm) to determine tick density per person-hour according to different times (Fig 2). Monthly rotations of collection points minimized location effects. Collection of questing ticks was conducted in grazing areas and around farms. However, in Kampong Speu, the collection was done only in the areas surrounding farms due to logistical difficulties.

In the laboratory, ticks were sorted based on location, collection period, host, and predilection sites. Species identification was performed under a stereomicroscope using species descriptions and identification keys developed in-house by the Medical and Veterinary Entomology Unit at Institut Pasteur du Cambodge [34–38].

### Statistical analysis

Tick density was expressed as the mean number of ticks per cow and the mean number of ticks per person-hour for questing tick collections, presented as mean ±  SD. To observe seasonality, tick density was analyzed according to four seasonal phases: (a) **early dry season** (November – January), characterized by low precipitation (mean daily: 2 mm) and moderatey cool weather with a mean temperature of 26.6°C (min: 23.1°C, max: 31°C); (b) **late dry season** (February – April), characterized by low precipitation (mean daily: 0.7 mm) and hot weather with a mean temperature of 29.8°C (min: 25.4°C, max: 35°C); (c) **early rainy season** (May – July), characterized by increasing precipitation (mean daily: 6.5 mm) and a mean temperature of 29.2°C (min: 26.2°C, max: 34°C); (d) **late rainy season** (August - October), characterized by relatively high precipitation (mean daily: 8.3 mm) and a mean temperature of 28.1°C (min: 25.2°C, max: 32°C).

The nonparametric Wilcoxon test was used to compare the average of tick infestation between female and male cattle. The Kruskal-Wallis test was used to compare the average of monthly collected ticks. Factorial analysis of variance (ANOVA) was used to test the impact of stage and predilection sites, including their interaction with time, for dragging and flagging data. Dunn’s test was used for pairwise comparisons of tick density per season, tick stage, and tick attachment across different cattle body parts. All statistical analyses were conducted using R Studio version 4.2.2. [39]. The infestation rate was calculated using the formula: (number of infested animals/total number of animals) x 100 [40].

## Results

### Tick collection according to host sex

Among the 240 animal hosts sampled across Kampong Speu and Takeo provinces, 210 cattle (87.5%) were found to be infested with ticks. In Kampong Speu, 100 females and 20 males cattle were sampled (83.3% female; and 16.7% male), while in Takeo province, 99 females and 21 males cattle were sampled (82.5% female and 17.5% male). In Kampong Speu, 77 out of 100 females cattle were infested with ticks, resulting in a prevalence of 77%, while 15 out of 20 males cattle were infested, giving a prevalence of 75%. This indicates no difference in tick infestation rates between female and male cattle, suggesting that infestation rates are similar across sexes in this province. In Takeo, tick infestation rates are higher in overall, with 98 out of 99 females cattle infested (0.99) and 20 out of 21 male cattle infested (0.95) (Table 1). There is also no difference of the infestation rate between male and female cattle in Takeo. Statistical analysis showed no significant difference in tick infestation rates between sexes in each province, with Wilcoxon tests showing p-values of 0.3 for Kampong Speu and p-values of 0.64 for Takeo.

**Table 1:** Prevalence of ticks according to sex of cattle.

Province	Sex	No. of cattle surveyed	No. of cattle infested	Prevalence %
Kampong Speu	Female	100	77	77.0
	Male	20	15	75.0
Takeo	Female	99	98	99.0
	Male	21	20	95.2

### Tick collection on host

A total of 13,678 ticks were collected in Kampong Speu (n =  9,087) and Takeo (n =  4,591). The overall average density of ticks per cow across both provinces was 56.8 ticks (56.8 ±  69.9). In details, cattle in Kampong Speu had in average 75.7 ticks per cow (75.7 ±  90.8), while in Takeo cattle had 38.3 ticks per cow (38.3 ±  30.9). The maximum number of ticks observed on a single cow was 570 ticks in Kampong Speu, and 183 ticks in Takeo.

Two species of ticks were identified at both locations: the *R. microplus* complex and *R. haemaphysaloides* (Table 2), with the vast majority being *R. microplus* complex (>99%). In Kampong Speu, an assessment of tick infestations among 120 cattle showed significant findings across different species. A total of 5,489 *R. microplus* complex were collected from 89 cattle, yielding in a density per cow (45.7 ±  55.8) following by *Rhipicephalus* spp. accounted for 3,453 ticks on 80 cattle (29.5 ±  46.5). Furthermore, *R. haemaphysaloides* exhibited a notably lower presence, with only 55 ticks collected from 32 infested cattle, with a lowest density per cow (0.45 ±  0.95). In Takeo, similar observations were collected: a total of 2,797 *R. microplus* complex on 118 cattle with the highest density per cow (23.3 ±  19.4) followed by *Rhipicephalus* spp. 1,791 on 109 cattle (14.9 ±  15.2) and only three *R. haemaphysaloides* were collected from three cattle, showing a very low density per cow (0.02 ±  0.15) (Table 2).

**Table 2:** Prevalence of ticks on cattle from Kampong Speu and Takeo, Cambodia.

Host (n total inspected)	Tick species	n cattle infested (%)	Total number of ticks	Density per cow (Mean ± SD)
**Kampong Speu**				
* Bos indicus* (n = 120)	*Rhipicephalus microplus* complex	89 (74.1)	5,489	45.7 ± 55.8
	*Rhipicephalus haemaphysaloides*	32 (26.6)	55	0.45 ± 0.95
	*Rhipicephalus* spp.	80 (66.6)	3,543	29.5 ± 46.5
**Takeo**				
* Bos indicus* (n = 120)	*Rhipicephalus microplus* complex	118 (98.3)	2,797	23.3 ± 19.4
	*Rhipicephalus haemaphysaloides*	3 (2.5)	3	0.02 ± 0.15
	*Rhipicephalus* spp.	109 (90.8)	1,791	14.9 ± 15.2
		210 (87.5)	13,678	

### Seasonality of *Rhipicephalus microplus* complex infestation on cattle

**Fig 3:**
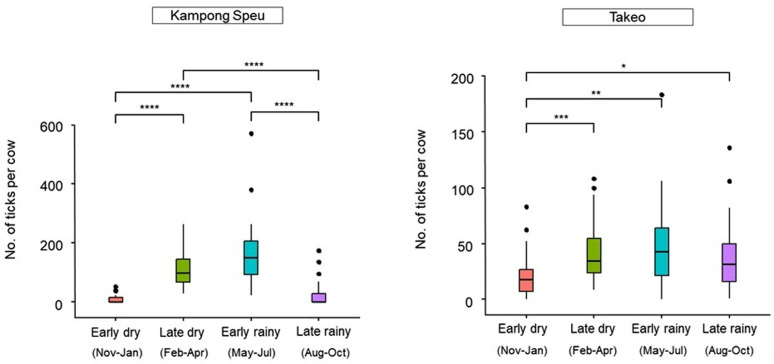
Temporal distribution of tick infestation on cattle over one year period by season.

The results revealed a significant seasonal effect on tick density at both Kampong Speu and Takeo farms (Fig 3). The Kruskal-Wallis test showed significant seasonal variation in tick density in Kampong Speu (χ² =  74.2, df =  3, p =  5.38e-16), and in Takeo (χ² =  18.22, df =  3, p =  3.95e-4). In Kampong Speu, tick density began to increase in the early dry season, with 294 ticks collected (9.8 ±  15.8). This increase continued into the late dry season, with a sharp rise to 3,319 ticks (111.0 ±  57.3). The peak tick density occurred in the early rainy season, with 4,759 ticks collected (159.0 ±  112.0). Tick density then declined in the late rainy season, with 715 ticks collected (23.8 ±  44.7). Similarly, in Takeo, tick density also started to rise in the early dry season, with 633 ticks collected (21.1 ±  18.8). This trend persisted into the late dry season, where tick numbers increased to 1,337 (44.6 ±  27.8). The highest tick density in Takeo was observed in the early rainy season, with 1,411 ticks collected (47.0 ±  36.8). Tick density then decreased in the late rainy season, with 1,210 ticks collected (40.3 ±  31.9). These findings the seasonal fluctuations in tick density, with peaks during the early rainy season and notable decreases during the late rainy season.

### *Rhipicephalus microplus* complex infestation according to life stages

**Fig 4:**
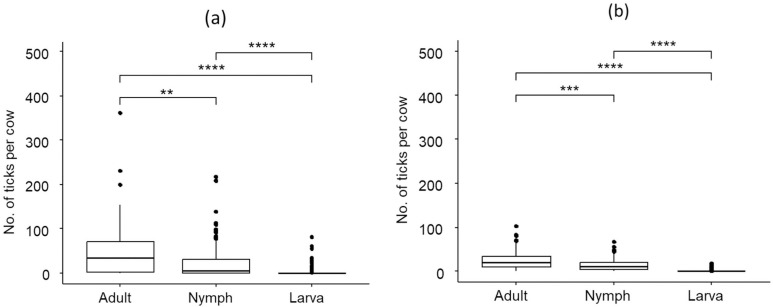
Prevalence of tick infestation on cattle based on life stages in (a) Kampong Speu and (b) Takeo.

Tick infestation across life stages on cattle in Kampong Speu (Kruskal-Wallis test, χ² = 78.6, df = 2, p <  0.0001) and Takeo provinces exhibited significant differences (Kruskal-Wallis test, χ² = 209.52, df = 2, p <  0.0001) (Fig 4). In Kampong Speu, adult ticks were the most prevalent (46.2 ±  56.6), followed by nymphs (24.0 ±  39.2) and larvae (5.06 ±  13.0). Similarly, in Takeo, adult ticks were predominant (23.7 ±  19.7), followed by nymphs (13.8 ±  13.7) and larvae (0.75 ±  2.75). These results highlight significant differences in tick life stage distribution in cattle in the two provinces.

The analysis based on life stages revealed the seasonality of ticks, as indicated by a Kruskal-Wallis test. In Kampong Speu province, adult tick density increased from the early dry season, peaked in the early rainy season, and decreased in the late rainy season (S1 Fig). Similarly, in Takeo province, adult tick density increased from the early dry season to the late dry season, but with no significant differences observed in the subsequent seasons (S4 Fig). Nymph ticks in both Kampong Speu (S2 Fig) and Takeo (S4 Fig) exhibited a similar pattern, with density rising during the early dry season, peaking in the early rainy season, and showing no significant differences in the later seasons. Larvae ticks in Kampong Speu followed the same seasonal trend as adults, with density increasing during the early dry season, peaking in the early rainy season, and decreasing in the late rainy season (S3 Fig). In contrast, larvae tick density in Takeo showed no significant variation across seasons (S6 Fig).

### *Rhipicephalus microplus* complex infestation according to predilection sites

The pattern of tick distribution on various body parts showed significant differences in Kampong Speu (Kruskal-Wallis test: p = 1.1e-09) and Takeo provinces (Kruskal-Wallis test: p =  < 2e-16). In Kampong Speu, the ear was the most frequently infested body part across all tick stages on cattle, followed by the neck, legs, back, abdomen, tail, and lowest the head. Similarly, in Takeo, the ear was predominantly infested, followed by the legs, abdomen, neck, back, tail, and lowest on the head. These findings highlight the most common feeding sites for ticks were the ear and lowest on the head in both provinces (Table 3).

**Table 3:** Ticks abundance on cattle based on predilection sites.

Province	Predilection site	Adult		Nymph		Larvae	
		Total	Mean ± SD	Total	Mean ± SD	Total	Mean ± SD
**Kampong Speu**	Abdomen	860	7.16 ± 9.17^a^	287	2.39 ± 4.33^ab^	7	0.05 ± 0.32^a^
	Back	733	6.10 ± 11.93^a^	395	3.29 ± 6.04^ab^	32	0.26 ± 1.65^a^
	Ear	1,286	10.71 ± 23.38^a^	926	7.71 ± 16.72^a^	477	3.97 ± 11.94^b^
	Head	144	1.2 ± 2.59^b^	111	0.92 ± 2.18^b^	10	0.08 ± 0.45^a^
	Legs	952	7.93 ± 10.83^a^	275	2.29 ± 4.44^ab^	15	0.12 ± 0.94^a^
	Neck	923	7.69 ± 10.28^a^	569	4.74 ± 10.19^a^	27	0.22 ± 1.12^a^
	Tail	650	5.41 ± 10.90^a^	314	2.61 ± 6.61^ab^	39	0.32 ± 1.29^a^
**Takeo**	Abdomen	792	6.6 ± 6.80^a^	253	2.11 ± 2.81^a^	–	–
	Back	126	1.05 ± 3.4^b^	53	0.44 ± 0.95^b^	–	–
	Ear	635	5.29 ± 9.21^c^	538	4.48 ± 7.27^a^	75	0.62 ± 2.51^a^
	Head	25	0.20 ± 0.74^b^	12	0.10 ± 0.45^b^	–	–
	Legs	789	6.57 ± 7.11^a^	363	3.02 ± 4.18^a^	–	–
	Neck	419	3.49 ± 5.56^c^	373	3.10 ± 5.28^a^	15	0.12 ± 1.19^b^
	Tail	56	0.46 ± 1.12^b^	63	0.52 ± 1.27^b^	1	0.00 ± 0.09^b^

Dunn’s test: a # b, a = ab, a # b # c.

### Tick collected from dragging & flagging

A total of 11,384 ticks were collected from vegetation around the two farms. One *Rhipicephalus sanguineus* adult, five *Haemaphysalis* spp. nymph, 240 *Haemaphysalis* spp. larvae and 11,138 *Rhipicephalus* spp. (*Boophilus*) larvae were collected by dragging and flagging. In Kampong Speu, a total of 7,941 ticks were collected, yielding an average density of 221.0 ±  749.0 ticks per times of the day. The peak tick density of 1,484 per person-hour was observed around midday in April (n =  4,453), and lower densities of 0.33 ticks per person-hour were recorded in January, May, and November, these differences were not statistically significant (ANOVA test: p =  0.5) (S1 Table).

Similarly, in Takeo province, a total of 3,443 ticks were collected, resulting in an average density of 47.8 ±  147.0 ticks per times of the day. Notable a higher in the evening in the density of 658.0 ticks per person-hour in May (n = 987), and only one tick per person-hour on morning in July, these differences were also not statistically significant (ANOVA test: p =  0.7) (S1 Table).

Overall findings showed no significant differences in questing tick density on vegetation across different collection times, sites, and province (ANOVA test: p =  0.16).

## Discussion

According to a classification system used in Mexico [40] tick infestation level are categorized based on the number of ticks per animal: “low” for 1 to 30 ticks, “moderate” for 31 to 60 ticks, and “high” for more than 61 ticks. In this study, the overall average infestation level was considered as moderate with an average 57 tick per cow. In Kampong speu, the level is high, with an average of 75 ticks per cow, while Takeo is moderate, with an average of 38 ticks per cow. This represents the highest tick density per host animal (*Bos indicus*) compared to Thailand, where the average is 2.22 ticks per cow [41]. Moreover, a single cow in Kampong Speu experienced a severe infestation, with the tick count reaching 570 ticks in June.

Our study shows only two tick species from a *Rhipicephalus* genus were found infesting cattle in the study area: *Rhipicephalus microplus* complex and *R. haemaphysaloides*. Up to 99% of the total ticks collected on-host were identified as *Rhipicephalus microplus* complex in both provinces. In contrast, a notably higher species richness has been recorded in neighboring countries; three species in Thailand [22] and Indonesia [3], four species in Vietnam [24], Laos [42], and nine species in Sri Lanka [43]. In addition to the *Rhipicephalus* ticks, ticks from the genus *Haemaphysalis* and *Amblyomma* were recorded in Vietnam, Laos and Sri Lanka recorded. In our study, the collection is limited to only two cattle farms, therefore do not represent the diversity of ticks infesting cattle across different habitat types in the entire country. The finding of only two tick species could be attributed to several factors. The limited movement of cattle in the farm settings might restrict the introduction of ticks from other habitats, such as forests. Additionally, while the sampling effort was extensive, the study focused on only two cow farms in two locations with similar environmental conditions. This limited geographic scope could be a factor in the reduced diversity of tick species observed. Although additional species, such as *Rhipicephalus linnaei* “the tropical lineage of *R. sanguineus*” and *Haemaphysalis* spp., were collected from the vegetation surrounding the farm, their absence on cattle suggests that cattle are not their preferred hosts. *Rhipicephalus microplus* is known to be a predominant tick species on cattle in Thailand, Bangladesh, and Sri Lanka [41,43–45], consistent with the findings in Cambodia. Furthermore, higher prevalence of *R. microplu*s than any other species has been observed in India and Sri Lanka [43,46] with a reported prevalence of 63.33% in Pakistan [40,47], and 70.3% in Bhutan [48]. *Rhipicephalus microplus* is a complex species differentiated from the three tick species based on their morphological characteristics, consisting of *R. australis*, *R. annulatus*, and *R. microplus* (clades A, B, and C) [1]. The identification of this species complex presents challenges due to the similarity in morphology and discordance between morphology and molecular data, especially for *R. australis* and *R. microplus* [21,38]. Another species found infesting cattle in this study, *R. haemaphysaloides*, showed lower abundance and infestation rates compared to the *R. microplus* complex. It has a three-hosts life cycle, leading to lower host specificity [1], which may explain its lower abundance on cattle compared to *R. microplus* complex that almost exclusively infests Bovidae family. This species is known for its wider host preference, including domestic animals and wildlife, and also may serves as vector of pathogens [1,49]. This species has been found infesting pigs and rabbits in Sri Lanka [43] and is considered as a common tick species infesting companion animals in worldwide [27].

This study is the first in Cambodia to describe the seasonal variation of tick abundance on cattle. In Thailand, research indicates that tick numbers peak during summer followed by winter and lowest in rainy season [41]. Similarly, in Vietnam, ticks also reach their highest density in the summer [24]. In contrast, the number of ticks tends to peak during the rainy season in Pakistan [50]. This pattern is consistent with observations from Cameroon, where ticks are most abundant in rainy season and least abundant in dry season [51]. These variations highlight the impact of seasonal changes and geographic differences on local temperature and environmental conditions influence on tick prevalence [41]. Furthermore, In Brazil temperature is critical for tick activity and survival on cattle, with optimal conditions not more than 28°C. However, temperatures exceeding this can be detrimental to tick development. Conversely, when temperatures fall below 19 ˚C, ticks may enter diapause, effectively halting their development and activity [52]. In this study, the peak density of ticks observed during the early rainy season is a consequence of the substantial increase of population density during the preceding driest and hottest months. These meteorological conditions seem to favorize the multiplication of the ticks in Cambodia. Understanding these regional differences in tick seasonality can be crucial for farm owners. Raising awareness about periods of peak tick abundance can help farmers for implementing timely preventive measures to manage tick infestations more effectively.

Ticks collected from cattle in this study predominantly consisted of adult ticks, followed by nymphs and larvae. The finding was consistent with those reported in China [53]. Biologically, cattle ticks complete their life cycle on a single host. They can develop from larva to adult within 23 days, although this duration varies based on climatic conditions, species, and geographical location [54]. Notably, male adult ticks can remain attached and feed for longer periods than females, typically lasting up to 70 days [55]. This extended feeding duration increases the likelihood of collecting adult ticks compared to immature stages. Moreover, adult ticks are larger and more visible compared to the immature stages, which are smaller and often harder to detect especially larvae due to their size and the limited time available during collection. This limitation in collecting immature stages may underestimate their actual prevalence and distribution on cattle. Therefore, studies focusing on tick populations should consider employing more refined methodologies that effectively capture and differentiate between all life stages to provide a comprehensive understanding of tick population structure on cattle.

Regarding tick predilection sites on cattle, the ear, legs, neck, abdomen, back, and tail have been identified as the favorable location for tick attachment. In both study sites, ear was found to have the highest tick abundance for all life stages, while the head had the lowest. Similar research in Nigeria also identified the ear, neck, legs, and head as favorable site for ticks, mainly to escape predators such as entomophagous birds [56]. However, the same study also revealed that age, sex, cattle breed, and color may influence the distribution of tick in the cattle body. Ticks often prefer the hidden, less-hairy, and well-vascularized areas to facilitate feeding and protection from predator [57–59], which explain the high number of ticks observed in areas such as the ear and under the tail. This finding enabled targeted interventions and monitoring strategies to reduce tick-borne diseases in cattle.

Immature ticks, such as larvae and nymphs, are typically more abundant in sampling by using flagging and dragging [31]. The questing behavior of immature stages makes them more accessible to these common sampling methods, which collect ticks from vegetation and the environment. The smaller size of immature ticks allows them to move through vegetation and other microhabitats more easily, further increasing their chances of being sampled [60]. In contrast, adult ticks, which spend much of their life cycle attached to hosts, are less likely to be collected through these methods. This is particularly true for ticks like *Rhipicephalus microplus*, which follows a one-host life cycle on bovine cattle, reducing the likelihood of adult ticks being captured from vegetation during methods such as flagging and dragging, which primarily target ticks in the environment. Notably, we collected adult *Rhipicephalus linnaei* (the tropical lineage of *R. sanguineus*) and immature *Haemaphysalis* spp. from vegetation but did not find these ticks on cattle hosts. This discrepancy could be due to the host preferences of these tick species. For instance, *R. linnaei* is more commonly associated with dogs rather than cattle, which may explain why we did not find it on the cattle hosts during our sampling [61,62]. Moreover, *Haemaphysalis* species, which were found in the immature stages on vegetation, may not be able to be collected from cattle hosts in their immature form, as they typically require specific host or different habitats for development before attaching to hosts. Interestingly, our tick sampling over vegetation revealed no significant effect of season and times of the day on the number of questing ticks in both Kampong Speu and Takeo province. There were no seasonal fluctuations in the density of ticks collected from vegetation around the farm. This suggests that cattle on the farm face a consistent risk of tick infestation from the environment throughout the year. The steady availability of hosts, like cattle, provides a stable environment for ticks, supporting the idea that tick populations in the pasture remain constant over time. While temperature is a key factor influencing tick activity, with higher temperatures facilitating feeding and growth, extreme heat (>40°C) can lead to dehydration and increased mortality rates for ticks [63]. In regions with more distinct seasonal fluctuations, such as Europe, environmental factors like temperature and humidity play a significant role in influencing tick questing behavior [64,65]. During the hottest parts of the day, tick larvae may retreat into the soil during the hotter parts of the day and emerge in the cooler morning or evening hours to search for hosts [66]. Local microclimate conditions and farming practices are more likely have significant effect to the activity of questing ticks. For example, the presence of agricultural area likely provides shade and maintaining a more favorable conditions for tick activity, regardless of the season. Farming practice, such as spraying insecticide or as simple as grass cutting may also affect the spatial distribution of the questing ticks on the vegetation [67,68]. These environmental factors play a crucial role in shaping tick activity patterns, emphasizing the importance of considering microclimate variations when implementing tick control measures and monitoring strategies in different regions [66]. Consequently, the dynamic of tick density in both the animals and the pasture appears to be balanced, with environmental factors such as host availability and habitat conditions playing crucial roles in tick distribution. Although limited to only two cattle farms, this study provides initial insight into basic knowledge on tick biology in the farm setting in Cambodia. A longer observation period is necessary to address the full dynamics of tick infestation on cattle such as variability over times of the day, seasonal patterns, and factors influencing tick density. Expanding the study to include more cattle farms across various geographical areas and ecological settings, combined with pathogen screening, would allow for a broader assessment of tick diversity and tick-borne disease threats in Cambodia.

## Conclusions

The *Rhipicephalus microplus* complex shows the highest density complex among tick species infesting cattle in Cambodia, posing a significant threat to livestock due to high infestation rate. Effective vector control measures are essential to mitigate this risk. To optimize cattle health and productivity, preventive tick management should be prioritized at the onset of the dry season, before infestation peaks.

## Supporting information

S1 Table Density of *Rhipicephalus* spp. larvae collected from vegetation around cattle farm in both province. (TIF)

S1 Fig Seasonal fluctuations in the density of adult tick of *Rhipicephalus microplus* complex in Kampong Speu. (TIF)

S2 Fig Seasonal fluctuations in the density of nymph tick of *Rhipicephalus microplus* complex in Kampong Speu. (TIF)

S3 Fig Seasonal fluctuations in the density of larva tick of *Rhipicephalus microplus* complex in Kampong Speu. (TIF)

S4 Fig Seasonal fluctuations in the density of adult tick of *Rhipicephalus microplus* complex in Takeo. (TIF)

S5 Fig Seasonal fluctuations in the density of nymph tick of *Rhipicephalus microplus* complex in Takeo. (TIF)

S6 Fig Seasonal fluctuations in the density of larva tick of *Rhipicephalus microplus* complex in Takeo. (TIF)

S1 Dataset Dataset Longitudinal. (XLSX)
