# Supplementary figures and images for: Seasonal dynamic of ticks infesting cattle (Bos indicus) farms in two provinces in Cambodia
Source: PLoS One. 2025 Apr 16;20(4):e0320879. doi: 10.1371/journal.pone.0320879 (PMC12002459; doi:10.1371/journal.pone.0320879)

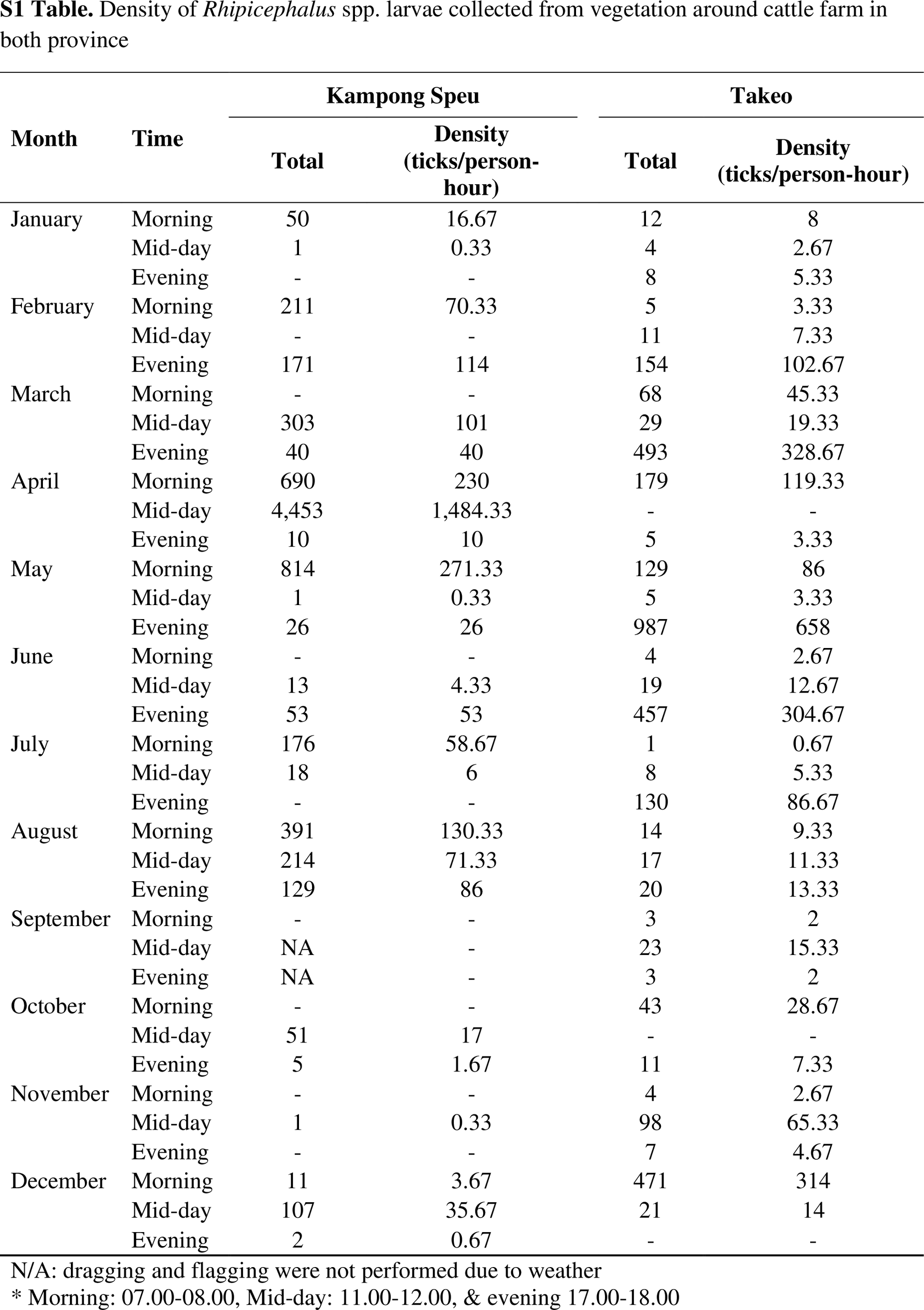

Supplement: S1 Table — (TIF) [file pone.0320879.s001.tif]

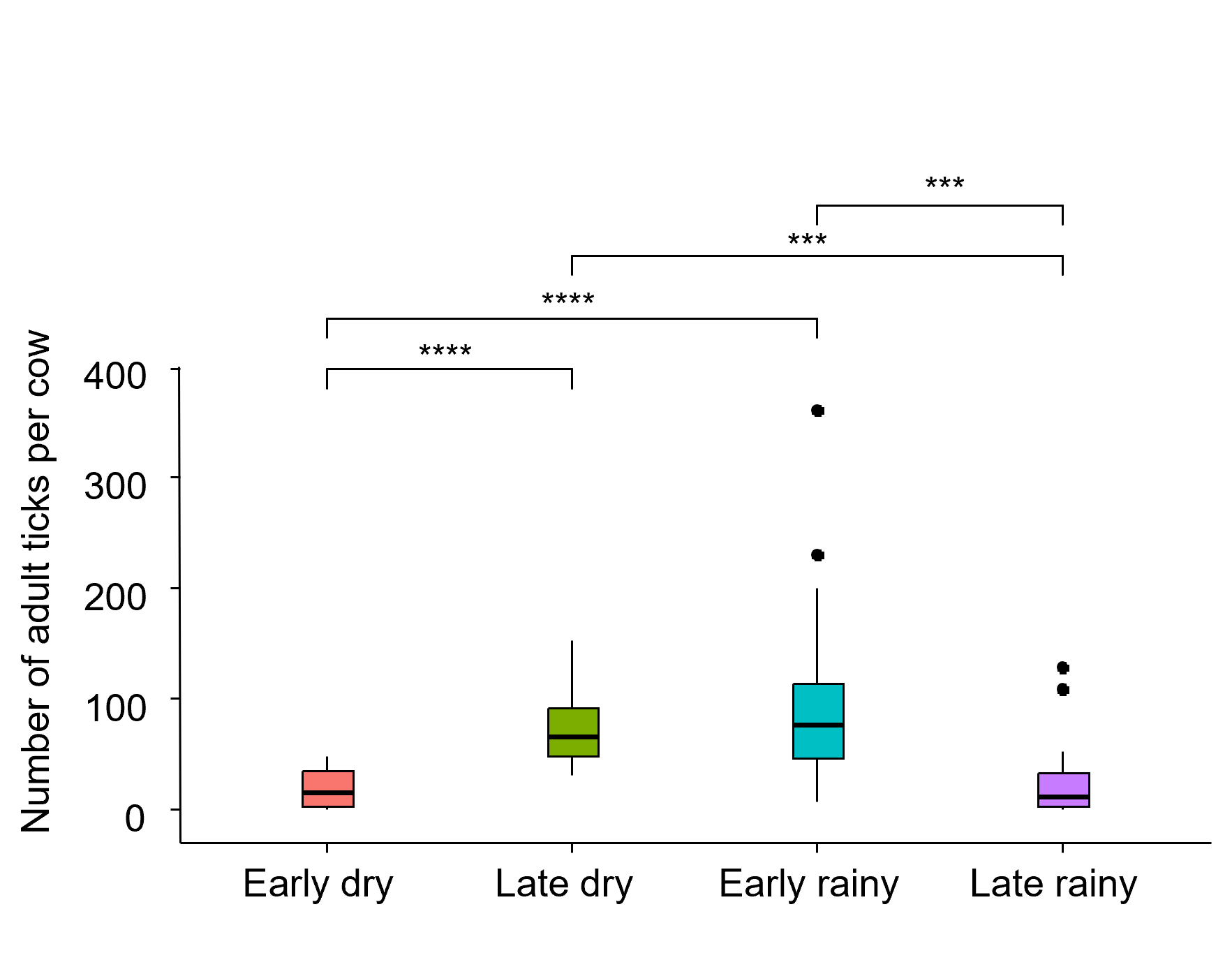

Supplement: S1 Fig — (TIF) [file pone.0320879.s002.tif]

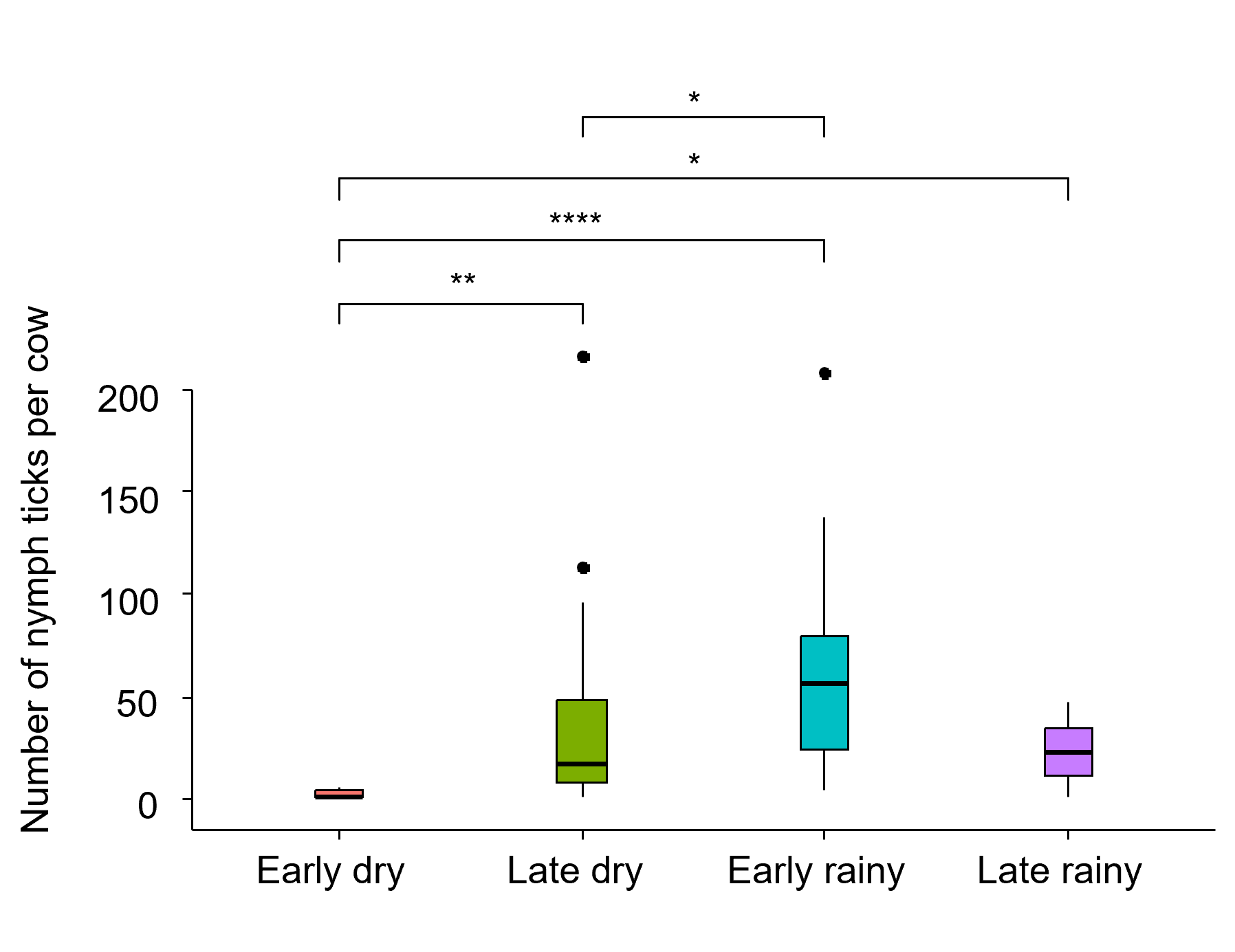

Supplement: S2 Fig — (TIF) [file pone.0320879.s003.tif]

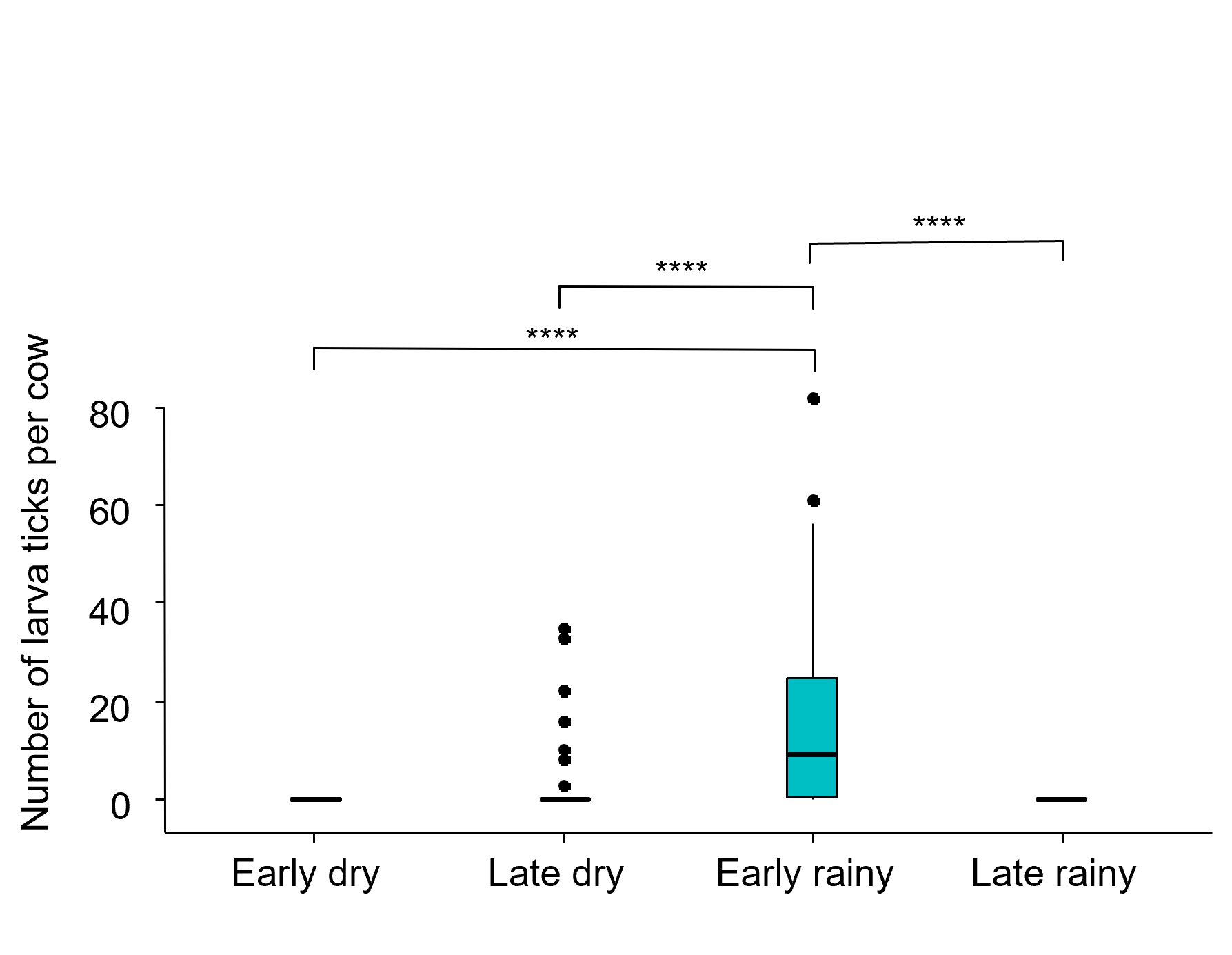

Supplement: S3 Fig — (TIF) [file pone.0320879.s004.tif]

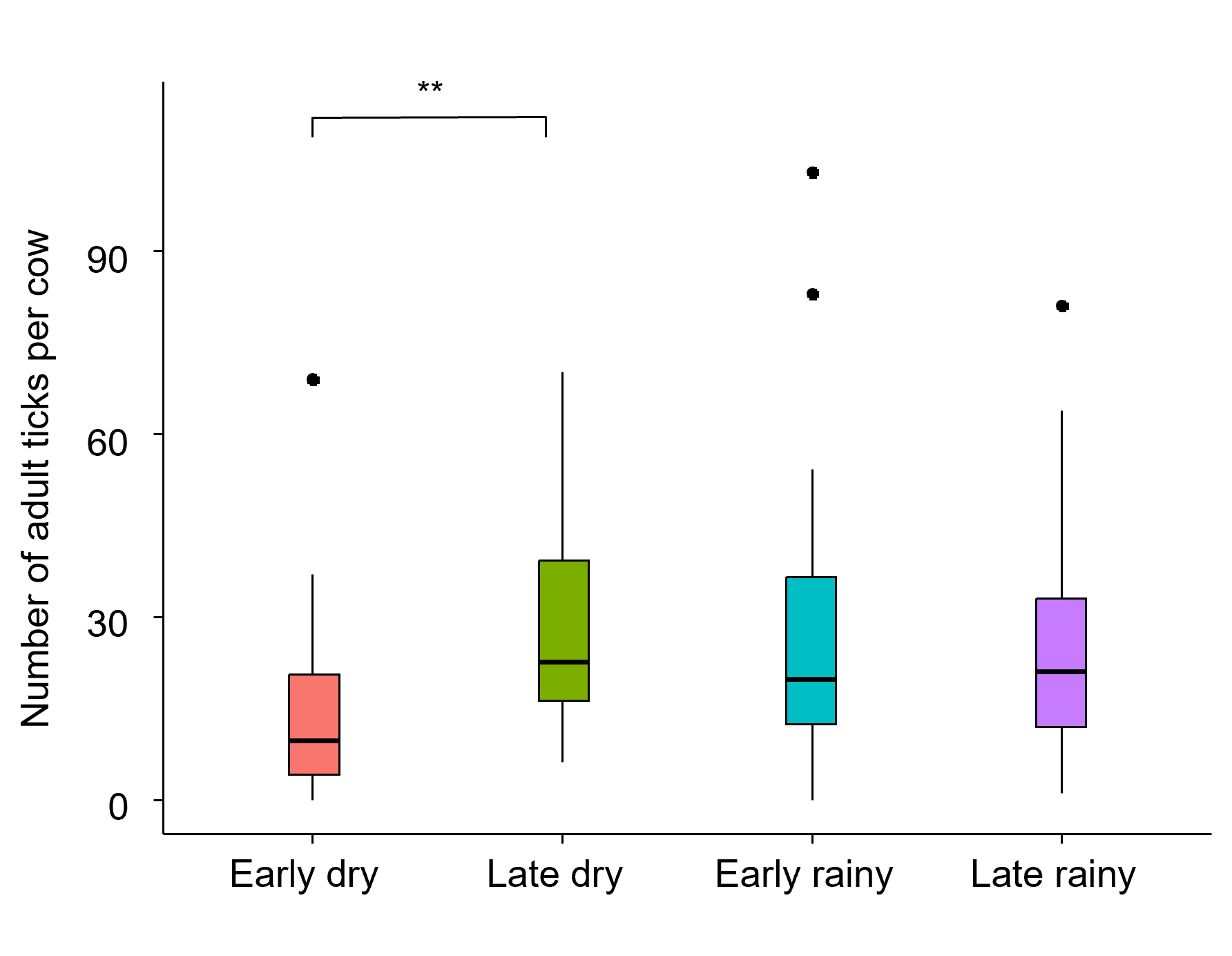

Supplement: S4 Fig — (TIF) [file pone.0320879.s005.tif]

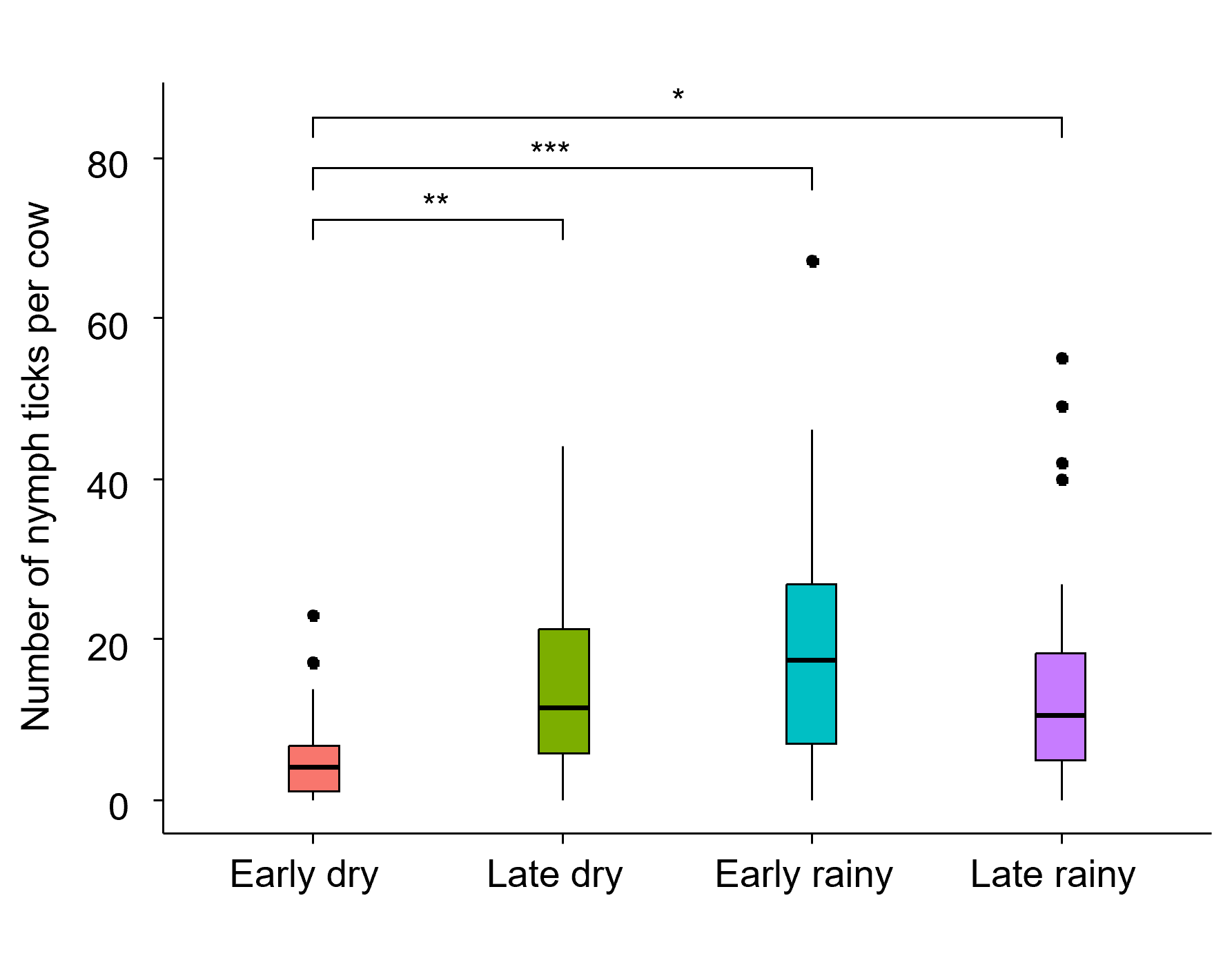

Supplement: S5 Fig — (TIF) [file pone.0320879.s006.tif]

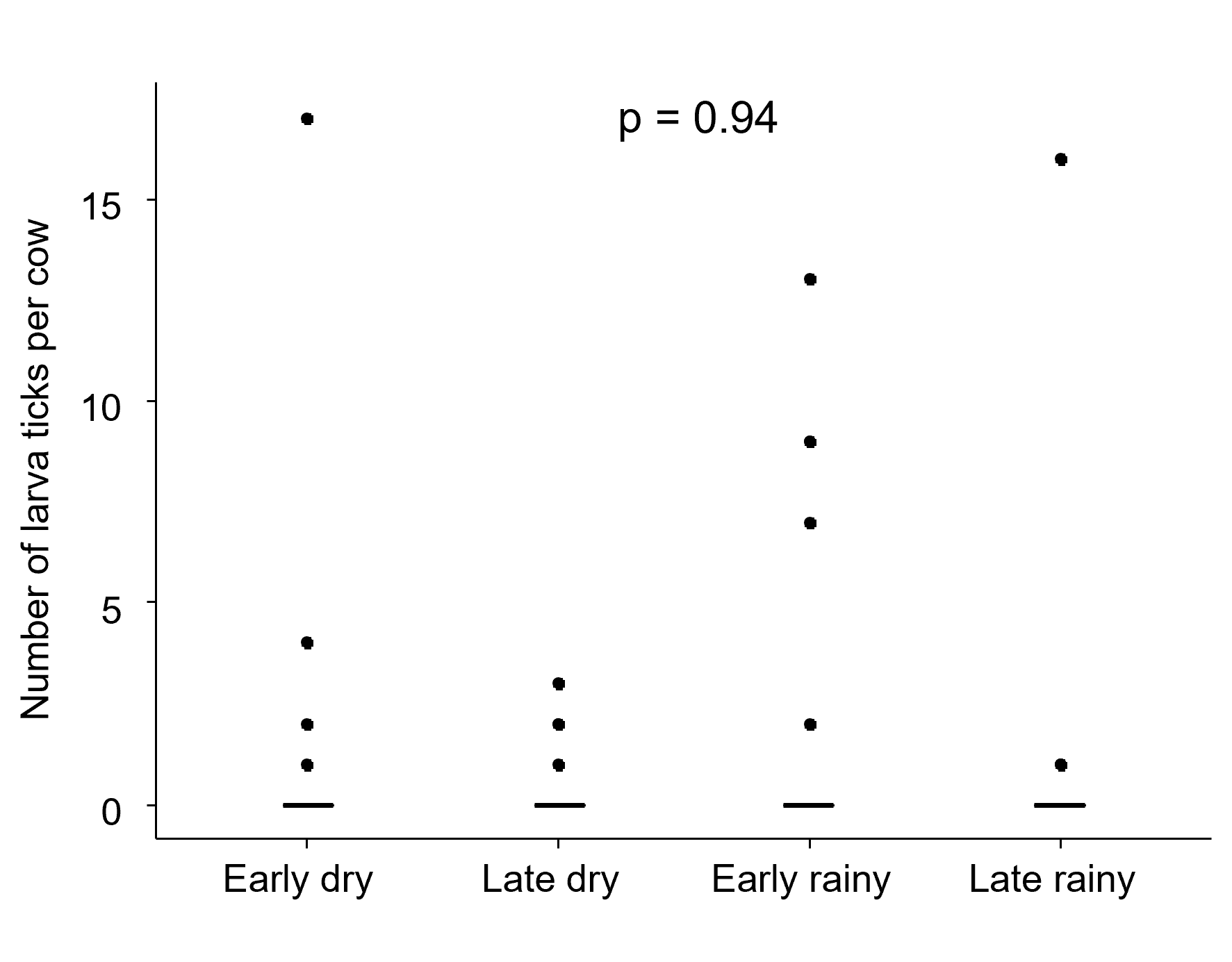

Supplement: S6 Fig — (TIF) [file pone.0320879.s007.tif]
